# Conventionally-fractionated image-guided intensity modulated radiotherapy (IG-IMRT): a safe and effective treatment for cancer spinal metastasis

**DOI:** 10.1186/1748-717X-3-11

**Published:** 2008-04-22

**Authors:** Youling Gong, Jin Wang, Sen Bai, Xiaoqin Jiang, Feng Xu

**Affiliations:** 1 State Key Laboratory of Biotherapy, West China Hospital, Sichuan University, Chengdu 610041, Sichuan Province, PR. China; 2 Department of Thoracic Oncology, Tumor Center, West China Hospital, Sichuan University, Chengdu 610041, Sichuan Province, PR. China; 3 Radiation&Physics Center, Tumor Center, West China Hospital, Sichuan University, Chengdu 610041, Sichuan Province, PR. China; 4 Department of Abdominal Oncology, Tumor Center, West China Hospital, Sichuan University, Chengdu 610041, Sichuan Province, PR. China

## Abstract

**Background:**

Treatments for cancer spinal metastasis were always palliative. This study was conducted to investigate the safety and effectiveness of IG-IMRT for these patients.

**Methods:**

10 metastatic lesions were treated with conventionally-fractionated IG-IMRT. Daily kilovoltage cone-beam computed tomography (kV-CBCT) scan was applied to ensure accurate positioning. Plans were evaluated by the dose-volume histogram (DVH) analysis.

**Results:**

Before set-up correction, the positioning errors in the left-right (LR), superior-inferior (SI) and anterior-posterior (AP) axes were 0.3 ± 3.2, 0.4 ± 4.5 and -0.2 ± 3.9 mm, respectively. After repositioning, those errors were 0.1 ± 0.7, 0 ± 0.8 and 0 ± 0.7 mm, respectively. The systematic/random uncertainties ranged 1.4–2.3/3.0–4.1 before and 0.1–0.2/0.7–0.8 mm after online set-up correction. In the original IMRT plans, the average dose of the planning target volume (PTV) was 61.9 Gy, with the spinal cord dose less than 49 Gy. Compared to the simulated PTVs based on the pre-correction CBCT, the average volume reduction of PTVs was 42.3% after online correction. Also, organ at risk (OAR) all benefited from CBCT-based set-up correction and had significant dose reduction with IGRT technique. Clinically, most patients had prompt pain relief within one month of treatment. There was no radiation-induced toxicity detected clinically during a median follow-up of 15.6 months.

**Conclusion:**

IG-IMRT provides a new approach to treat cancer spinal metastasis. The precise positioning ensures the implementation of optimal IMRT plan, satisfying both the dose escalation of tumor targets and the radiation tolerance of spinal cord. It might benefit the cancer patient with spinal metastasis.

## Background

Spine is the most common place of cancer metastasis, especially for lung cancer and breast cancer. Each year, approximately 50,000 patients with cancer develop spinal metastasis worldwide and the 5-year over-all survival rate of these patients was less than 5% [1,2]. All together, accompanying with the improvement of therapy for malignant tumors, the overall survival time of cancer patients prolonged and the incidence of spinal metastasis was increasing gradually. Radiotherapy is the standard treatment for vertebral metastasis of patients with cancer. Reviewing the literatures, three treatments/fractions were applied clinically worldwide: 30 Gy/10 fractions, 20 Gy/5 fractions and 8 Gy/1 fraction [3,4]. But all three treatments were palliative, and recurrences in pre-irradiated foci were frequent. Especially for those patients who only had vertebral metastasis with primary lesion controlled, higher dose may increase the local control and survival possibility of such patients.

To avoid radiation necrosis, the conventionally-fractionated radiotherapy always prescribed no more than 50 Gy on metastatic sites that were often insufficient to achieve acceptable local disease control and only inhibit tumor growth. The more conformal dose distribution of intensity-modulated radiation therapy (IMRT) may provide satisfactory dose coverage of tumor and avoid excessive radiation of surrounding normal tissue, therefore with potential advantage to achieve higher therapeutic ratio. However the vertebral metastasis was often adjacent to spinal cord and the sharp dose gradients between PTV and spinal cord requires high precision of daily positioning to guarantee implementation of IMRT. Without special techniques that allow highly accurate set-up and dose escalation, some patients who might benefit from radiotherapy may remain untreated or may be treated with doses unlikely to provide long-term local control.

So far, surgery is usually offered to patients with a reasonable life expectancy, whose spinal instability was present and was causing symptoms [5,6]. Surgery also has been used for more aggressive and relatively radio-resistant tumors. Also, the stereotactic radiosurgery is another choice for those patients. A few study reported that the single- or hypo-fractionated radiosurgery had the promising results in the treatment for cancer spinal metastasis [7-10]. But in practice, the treatment failures were still common [11,12].

To date, no ideal treatment could be prescribed for these cancer patients. The newly developed Elekta Synergy™ is an integrated image-guided radiotherapy (IGRT) system with the kV-CBCT system attached to a digitalized medical linear accelerator that can provide onboard CBCT imaging of set-up errors. Thus, it had been stated as a potential treatment for cancer spinal metastasis. In this paper, we report the preliminary results of the application with this technique, giving details about the safety and effectiveness of IMRT dose escalation with IGRT for metastatic tumors of the spinal vertebra.

## Methods

### Patient selection

This study was carried out in Tumor Center at West China Hospital, Sichuan University, PR. China. Between May and November 2006, 9 previously treated cancer patients with confirmed diagnosis of ≤ 2 spinal metastases and no other distant metastasis were recruited in this study. The basic and clinical characteristics of these patients were shown in Table 1. Each diagnosis was confirmed by computed tomography (CT), magnetic resonance imaging (MRI) or positron emission tomography-CT (PET-CT) before the treatment. And KPS scores of the patients were ≥ 80 when admitted in our hospital, with life expectancy of more than 6 months. This study was carried out with the approval of West China Hospital's ethics committee.

**Table 1:** Basic and clinical characteristics of the study population (n = 9)

**Age (years)**	
<45	4
≥ 45	5
**Gender**	
Male	4
Female	5
**Cancer type**	
Lung cancer	2
Breast cancer	3
Colorectal cancer	2
Other cancer types	2
**Spinal metastasis site(n = 10)**	
Cervical	2
Thoracic	5
Lumbar	3
**Total volume of GTV (mm3)**	
≤ 20	2
20 ~ 40	6
≥ 40	2

### Treatment planning and evaluation

Each patient underwent spiral CT simulation with 3-mm slice thickness with vacuum mattress (Stereotactic Body Frame, Elekta, UK) immobilization. Target volumes and normal structures were contoured by radiation physicians. The gross target volume (GTV) represented areas at cancer metastatic parts of vertebra based on pre-planning CT, MRI or PET-CT imaging. If the whole vertebra was involved, the clinical tumor volume (CTV) was defined as equal to GTV; otherwise a 10 mm margin around GTV was added to generate CTV. For PTV, a 3 mm margin was added isotropically to CTV, and the PTV was not allowed to overlap with the adjacent spinal cord but could touch it. The spinal canal was contoured as a critical structure and to extend 2 cm length in SI direction beyond the level of PTV, with a median length of 11.6 cm (range of 8.1–13.4 cm) in planning. Depending on the metastatic sites, the lung, right/left kidney, and liver were delineated as other OAR. All target delineations were reviewed by three physicians and brought to the final consensus. The IMRT plan was generated using 9–12 axial beam angles using aperture-based inverse planning system (PrecisePLAN Release 2.11, Electa, Sweden). A dose of 60–64 Gy was prescribed to PTV in 29–31 fractions, and the planning was to deliver the prescribed dose to at least 95% of the PTV with a dose range not exceeding -10% and +15% of the prescribed dose. The dose to spinal cord was restricted within 50 Gy. The minimum segment size was 2 cm^2 ^with a minimum of 4 monitor units (MU), a median of 43 (35–55) segments were planned. Segments were manually adjusted after aperture-based optimization to increase the dose gradient between target and OAR in 3 patients.

All plans were evaluated according to DVH analysis. The homogeneity index (HI) was defined as D_5_/D_95 _(minimum dose in 5% of the PTV/minimum dose in 95% of the PTV). The lower (closer to 1) the HI is, the better the dose homogeneity. Also, the conformity index (CI) was calculated as follows: CI = CF (cover factor)·SF (spill factor), where the CF was defined as the percentage of the PTV volume receiving the prescription dose and the SF as the volume of the PTV receiving the prescription dose relative to the total prescription dose-volume (see also RTOG protocol 9803). The closer the CI value to approach 1, the better the dose conformity is.

IMRT plan was delivered with step-and-shoot technique utilizing the system's Beam Modulator™ that is an 80-leaf MLC with a leaf width of 4 mm (at the isocenter).

### KV-CBCT imaging

Daily kV-CBCT images were acquired with the VolumeView™ XVI function. The XVI allows acquiring a series of projected images at different gantry rotation that can be reconstructed to 3-dimensional volumetric data, cut to sections and registered to input planning CT for matching. The parameters for CBCT scan were 100–120 kV, scan started from 182–260° and ended at 100–180° with the total imaging dose of 16 mGy per scanning [13], utilizing medium resolution reconstruction. Each acquisition procedure (including image reconstruction) lasted 5 minutes. Daily CBCT images were registered with the planning CT using automatically bone matching (correlation coefficient algorithm, Elekta XVI software) to calculate the target deviations on the LR, SI and AP axis. The ROI for image registration was limited to the vertebrae on the level of the PTV. An action level of 2 mm was set for online correction of translational error. Only the translational errors of the target which exceed the 2 mm action limit were converted to a respective shift of the treatment table by manual adjustment. Rotational set-up errors were identified but unable to correct due to limitation of couch movement. If the rotational set-up errors exceed 2°, patient should be re-positioned immediately. CBCT re-scan should be applied to ensure action level not exceeded. The projection of isocenter was marked on the abdominal skin of each patient to verify the maintenance of patient set-up accuracy during treatment at the first fraction, and the patient set-up remained unmovable during the whole treatment.

The positioning errors were analyzed as described previously [14]: The mean of all displacements and the standard deviation (SD) of all displacements of the whole group of patients were calculated. For each patient individually the mean (systematic error) and standard deviation (random error) of all errors were calculated. The systematic uncertainty Σ is defined as the standard deviation of the systematic errors. The root-mean-square of the random errors was calculated as σ. Errors were calculated separately for all three axes (LR, SI and AP).

### Simulation of observed patient set-up errors

According to Yan et al [15], PTV margin can be designed based on a large confidence level (≥ 98%) with a simple recipe of 2.27 × SD. The margins based on initial set-up errors and post-correction errors were thus calculated. Then the calculated PTV margin at initial setup was added to CTV in three directions in each IMRT plans respectively, to generate another PTV (PTV_pre_) when no online correction was applied. To simulate the impact of online correction on dosimetry, the isocenter of the original IMRT plan was shifted towards each OAR with a magnitude that was equal to the difference between the calculated pre-correction margin and actual applied margin (3 mm). The dose-volume parameters of OARs of the original and simulated IMRT plans were then compared.

### Follow-up

Chemotherapy was prescribed after IG-IMRT. And patients were seen 1 month, and every 3 months after treatment. The 100 mm Visual Analog Scale (VAS) measure was used to evaluate the pain of these patients. The radiation-induced toxicities were assessed with RTOG criteria [16]. The median follow-up of the study patients was 15.6 months (range of 11–19 months).

## Results

**Figure 1:**
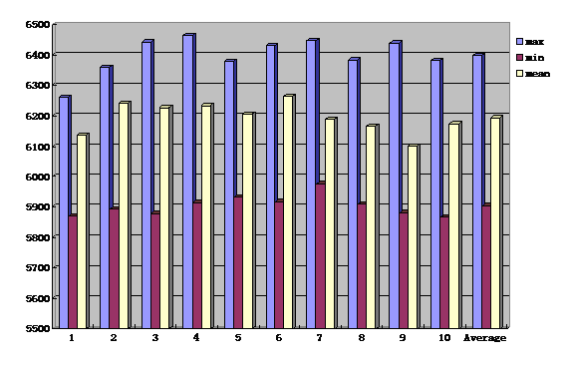
The maximum, minimum and mean dose of the 10 metastatic lesions (PTV) in treatment plans and the average level.

**Figure 2:**
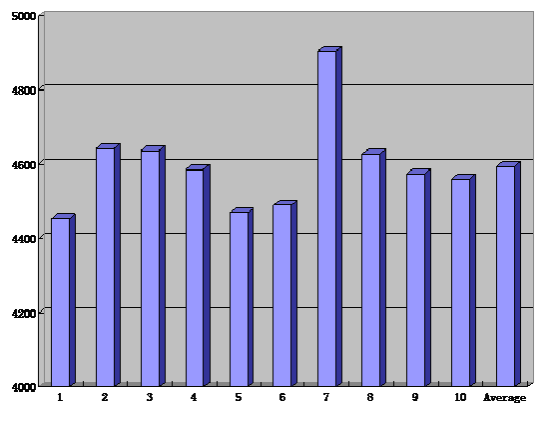
The maximum dose of the spinal cord in treatment plans and the average level.

**Figure 3:**
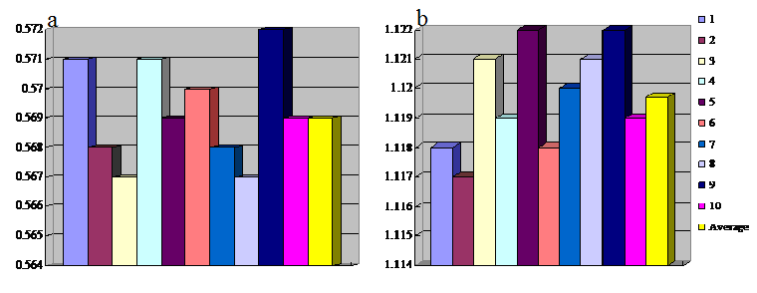
The homogeneity index/dose conformity index and the average level in treatment plans (1, 2, 3..10 represented the number of the IMRT plans, respectively).

**Figure 4:**
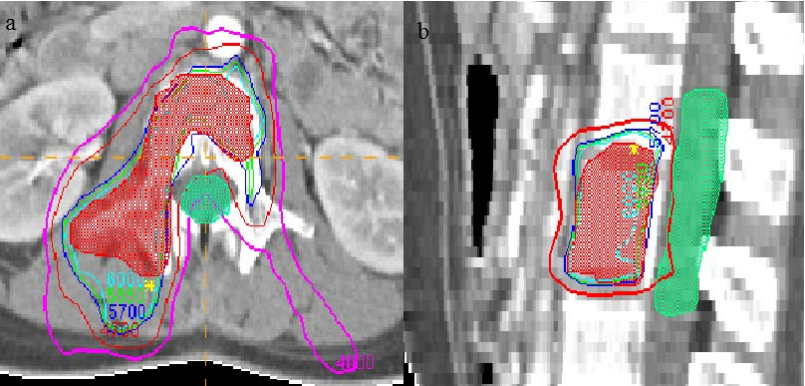
**A representative IMRT plan with radiation isodose curves.** The PTV (red region) was covered by the 95% curve of the prescription dose (the green line), and the dose of the adjacent cord was less than 49 Gy. (a: transverse section and b: sagittal section).

In treatment planning, the average dose which the PTVs received was 61.9 Gy, with the maximum dose of 64.6 Gy and the minimum dose of 58.7 Gy (Figure 1). The average level of the maximum dose which the adjacent cord received was 45.9 Gy, with a range of 44.5–49.0 Gy (Figure 2). Based on the DVH analysis, the average CI was 0.569, with a range of 0.567–0.572 (Figure 3). For HI, the maximum and the minimum values were 1.122 and 1.117 respectively, with an average value of 1.12 (Figure 3). A representative IMRT plan with radiation isodose curves was shown in Figure 4. The PTV (red region) was covered by the 95% curve (58.5 Gy, the green line) of the prescription dose (60 Gy), and the curve of 47 Gy touched the adjacent cord.

As shown in Table 2, both systematic (Σ) and random (σ) uncertainties were markedly reduced after online correction which ranged 0.1–0.2/0.7–0.8 mm after correction compared to 1.4–2.3/3.0–4.1 mm before correction. And the group mean (M) of the setup errors were small both before and after correction.

**Table 2:** The positioning errors before/after (without/with) online set-up correction in the LR, SI and AP axes in this study (mm)

	**LR**		**SI**		**AP**	
	
	Before	After	Before	After	Before	After
**mean**	0.3	0.1	0.4	0.0	-0.2	0.0
**SD**	3.2	0.7	4.5	0.8	3.9	0.7
**Range**	-12.0 ~ 13.5	-2.6 ~ 1.4	-17.2 ~ 16.3	-2.5 ~ 1.5	-12.9 ~ 10.9	-1.9 ~ 1.5
**Σ**	1.4	0.2	2.1	0.2	2.3	0.1
**σ**	3.0	0.8	4.1	0.7	3.2	0.7
**Theoretic margin**	7.4	1.7	10.2	1.6	8.8	1.7
**Translational shift**	4.4		7.2		5.8		

Before: before online set-up correction; After: after online set-up correction; Theoretic margin: calculated by 2.27 × SD based on a pre-selected confidence level of 98%; Translational shift: translational shift of the treatment isocenter in simulated IMRT plan to cover the theoretic margin without online set-up correction in three axes, and calculated as "theoretic margin before online set-up correction-3" mm.

According to the calculated pre-correction margins (2.27 × SD) shown in Table 2, the volume of the actual PTV (PTV_real_) in the applied IMRT plans and simulated PTV_pre _were shown in Table 3 in details. The average volume of PTV_real _and PTV_pre _was 77.1 and 133.7 cm^3 ^respectively; with an average reduction of 42.3% after online correction. The impact of translational shift of treatment isocenter towards each OAR on the dose-volume parameters was shown in Table 4. More notably, the average reduction in dose-volume parameters of OAR from PTV_pre _to PTV_real _were 14.8%, 10.7% and 14.5% in the mean dose, V_20 _and V_12.5 _of the lungs; 19.9%, 33.3%, 29.6% and 21.1% in the mean dose, V_30_, V_20 _and V_10 _of the liver; 21.9%, 42.9%, 23.8% and 20.5% in the mean dose, V_30_, V_20 _and V_10 _of the right/left kidney; 28.2% and 16.7% in the maximum dose and D_5_^spine ^(maximum dose in 5% volume of the spinal cord) of spinal cord, respectively.

**Table 3:** The volumes of original and simulated PTVs in this study (cm^3^)

**Target number**	**PTVreal**	**PTVpre**	**Volume reduction from PTVpre to PTVreal (%)**
1	15.6	26.4	40.9%
2	94.7	168.6	43.8%
3	82.5	126.2	34.6%
4	89.3	150.6	40.7%
5	72.6	116.4	37.6%
6	18.9	37.2	49.2%
7	108.4	204.3	46.9%
8	91.2	155.8	41.4%
9	112.3	221.2	49.2%
10	85.9	130.6	34.2%
Average	77.1	133.7	42.3%

PTVreal: actual PTV in the original IMRT plans; PTVpre: simulated PTV based on the theoretic margins in three axes without online set-up correction.

**Table 4:** Average normal tissue dose-volume parameters based on PTVpre and PTVreal in each original and simulated IMRT plans

Normal tissue parameter	Average parameters based on PTVpre	Average parameters based on PTVreal	Average parameters reductions from PTVpre to PTVreal (%)
**Lung (n = 4)**			
Maximum dose	57.3 Gy	55.2 Gy	3.7
Average dose	10.8 Gy	9.2 Gy	14.8
V20	12.2%	10.9%	10.7
V12.5	23.5%	20.1%	14.5
**Liver (n = 4)**			
Maximum dose	58.6 Gy	56.1 Gy	4.3
Average dose	14.6 Gy	11.7 Gy	19.9
V30	12%	8%	33.3
V20	27%	19%	29.6
V10	38%	30%	21.1
**kidney (n = 4)**			
Maximum dose	60.2 Gy	57.8 Gy	4.0
Average dose	14.6 Gy	11.4 Gy	21.9
V30	7%	4%	42.9
V20	21%	16%	23.8
V10	39%	31%	20.5
**Cord (n = 10)**			
Maximum dose	68.4 Gy	49.1 Gy	28.2
Average dose	34.3 Gy	31.2 Gy	9.1
D5spine	54.4 Gy	45.3 Gy	16.7

PTVpre: simulated PTV based on the theoretic margins in the three axes without online set-up correction; PTVreal: actual PTV in the original IMRT plans; D5spine: maximum dose in 5% volume of the spinal cord.

Clinically, grade 1/2 acute radiation-induced skin toxicity was observed during treatment, and the majority of patients had prompt pain relief within 4 weeks of treatment. According to their VAS score, the average level was 83 mm (range, 70–90 mm) at the baseline. 4 weeks after IG-IMRT, the average score decreased to 52 mm, with a range of 40–62 mm. And at the end of follow-up, the average VAS score of these patients was 42 mm. 3 months after treatment, one patient developed progressive metastasis in the brain, and one developed liver metastasis, but the regions of the spine treated with IG-IMRT were clinically stable. No patient developed acute radiation-induced injury after the treatment. During follow-up, the lower extremity strength and ambulation of all patients remained stable and no patients have experienced complications as a result of the procedure.

## Discussion

The irradiation tolerance of the spinal cord, the TD_5/5_, is considered to be in the range of 50 Gy for single daily fractions of 1.8–2.0 Gy [17]. The dose required for cure of a cancer spinal metastasis should be analogous to that of the primary site, which generally should not be less than 60 Gy (1.8–2.0 Gy/fraction) for solid tumors. Obviously the standard conventionally-fractionated 30–40 Gy was insufficient for long-last control of the spinal metastasis, resulted in the infield failure to be 26% or more [18]. Several studies had been reported using single/hypo-fractionated radiosurgery for cancer spinal metastases [7-12]. According to the linear quadratic formula [19], the biological-effective-dose (BED) of the metastatic lesions received in these studies was between 60–153 Gy_10_. The clinical outcome indicated that radiosurgery was effective in the treatment of these patients, improving long-term palliation. However, the efficacy and safety of radiosurgery is limited by tumor volume and the closeness of targets to the critical organs, for larger tumors the dose is often reduced to avoid radiation-induced necrosis. Another limitation inherent of radiosurgery is that it delivers radiation over a single session and thus does not encounter multiple mitotic phases, which may spare the cells staying in the radioresistant phases and increases risk of recurrence especially with reduced dose [20]. Recently, improvement in radiation technique provides potential means of IMRT dose escalation for spinal metastasis cancer. Thus for the first time, conventionally-fractionated radiotherapy with daily CBCT online correction was applied for cancer spinal metastasis in this study: the BED was in a range of 97–107 Gy_10 _for the metastatic lesions and the irradiation dose of the spinal cord was less than 49 Gy in 29–31 fractions. Comparing to the data from radiosurgery, a therapeutic dose was prescribed for tumor target with IMRT plan, guaranteeing the irradiation tolerance of the spinal cord. Follow-up showed no patient suffering from the radiation-induced necrosis as a result of the treatment and all patients had varying degrees of pain relief. The average VAS scores of these patients were 83, 52 and 42 mm before, 4 weeks after IG-IMRT and at the end of follow-up, respectively. Complete pain relief was observed in 3 patients, and the remaining 6 patients were able to reduce pain medication. The result was similar with those from radiosurgery and more superior to the palliative radiotherapy in such patient. Clinically, the treatment was effective in the studied population.

Due to the steep dose gradients between metastatic lesions and spinal cord of the IMRT plan, very precise set-up procedure before radiotherapy is necessary. With the application of IGRT technique, patient set-up accuracy was verified by in-room CT scanner, helical tomotherapy, orthogonal X-ray cameras, and CT on rail in radiotherapy for spinal or paraspinal cancer [12,21-24]. Basically, simply applying the patient immobilizing technique with wall laser marks on the body surface still can not fulfill the stringent target position requirement of high precision radiotherapy. In this study, daily CBCT with online correction of set-up errors before treatment was practiced to achieve the maximum accuracy and safety for the patient. The systematic/random errors at initial set-up were 1.4/3.0, 2.1/4.1 and 2.3/3.2 mm in the LR, SI and AP axes, respectively. After set-up correction, those errors were 0.2/0.8, 0.2/0.7 and 0.1/0.7 mm in the three axes respectively, indicating the role of online correction on improving positioning precision for radiotherapy of spinal metastatic cancer, thus may potentially reduce the adverse effect of set-up errors on tumor control probability and normal tissue complication probability (NTCP) in radiotherapy treatment [25].

Based on the margin-calculating recipe, a 1.7, 1.6 and 1.7 mm margin should be added to the CTV for generating PTV in the LR, SI and AP axes respectively with CBCT online correction, confirming that the 3 mm region around the GTV/CTV was enough and acceptable with CBCT-based guidance. Without online correction, the calculated margins in the three axes were 7.4, 10.2 and 8.8 mm, respectively. In each IMRT plan, we simulated the hypothetic effects of the pre-correction positioning errors on PTV and dose-volume parameters of OAR. As shown in Table 3, the reduction of volume from the pre-correction PTV_pre _to the PTV_real _with online correction was considerable, with an average level of 42.3%. Also, the translational shift of isocenter towards each OAR had significant impact on the dose-volume parameters of these organs. Depending on the target location, there were 4 targets related to lung, 4 targets related liver and right/left kidney, and 10 targets related to spinal cord. The dose-volume parameters of each OAR were reduced to varying degrees. The dose reductions could translate clinically into a lower probability of treatment toxicity, as well as a potential increase in the number of patients that might be eligible for IG-IMRT or concurrent chemoradiotherapy.

**Figure 5:**
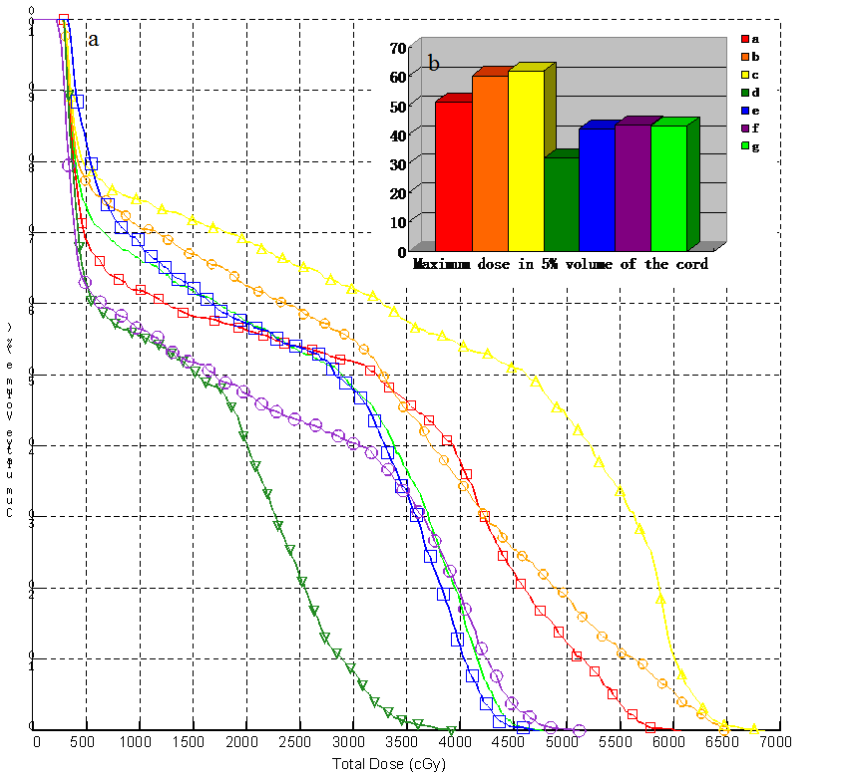
**Comparison of the simulated effects of the positioning errors with/without CBCT-based online set-up correction in the LR, SI and AP axes on the irradiation dose of the spinal cord with the actual plan (red and orange line: isocenter moving left/right in LR axis, yellow and deep green lines: isocenter moving superior/inferior in SI axis, blue and purple lines: isocenter moving anterior/posterior in AP axis, respectively; and the green line was the actual DVH of the cord).** (a: the simulated and actual DVHs of the cord and b: the simulated and actual maximum dose of 5% volume of the cord).

The spinal cord was the key OAR in this study. The isocenter was shifted in the six directions (moving left/right, inferior/superior, and anterior/posterior in LR, SI and AP axes) respectively to simulate the impact of pre-correction margin on the dose-volume parameters of the spinal cord. Figure 5 showed the simulated and original DVH of the spinal cord in one IMRT plan (the same patient as Figure 4 represented). The position errors in SI axes had little impact on the irradiation dose of the cord. As well, it indicated that the D_5_^spine ^changed significantly, if position errors occurred towards the cord in LR and AP axis, respectively. Most significantly, the posterior shift towards the cord resulted in a maximum dose of 68 Gy to the cord. Comparing to the results reported by Guckenberger *et al *[26], our study suggested that without the CBCT online guidance, the IMRT plan could not be applied successfully in such patients.

Although only the inter-fractional setup errors was taken into account as the major source of uncertainties to affect the accuracy of IMRT dose delivery in this study, there was another important factor which also contribute to the dose delivery accuracy: movement of the target and spinal cord during treatment (intra-fraction variation). First, CTV for the paraspinal lesions was assumed to be fixed to the vertebrae and the intra-treatment motion of the target would be equivalent to the motion of the spinal column. Data from literatures have confirmed that in conformal radiotherapy, intra-fraction organ/target motion can be achieved in the range of 1 mm with proper immobilization [21,27]. Second, Cai *et al *found that the spinal cord motion during normal breathing was typically within 0.5 mm by dynamic MRI (dMRI), and partly stated that the spinal cord was almost immovable during breathing [28]. Third, studies in Massachusetts General Hospital and Memorial Sloan-Kettering Cancer Center indicated that the effects of intra-fraction organ motion on IMRT dose delivery were ignorable in a typical treatment with 30 fractions in breast and pulmonary radiotherapy [29,30]. Obviously, for the more fixed OAR (spinal cord) in the vacuum mattress, the effects of intra-fraction organ motion would be more minimal in this conventionally-fractionated IMRT therapy. In addition, all target delineations were reviewed by three physicians together, diminishing the impact of the delineation-induced variation on the geometrical accuracy in conformal radiotherapy [31-33] as far as possible. Consequently, as discussed previously, the precise patient set-up with CBCT online correction was the minimal requirement and meaningful factor in dose delivery accuracy of IG-IMRT in this study.

Limitation in this study should be addressed here. The position errors should include not only the translational set-up errors, but also the rotational errors, which may have effect on the accuracy of dose delivery. A few studies evaluated the rotational set-up errors in conformal radiotherapy for spinal diseases [26,34]. Due to the limitation of the treatment couch, patient in our study should be re-positioned if the rotational set-up errors exceeded 2°. So, the rotational set-up errors and their impact on IMRT dose delivery had not been evaluated in this study.

## Conclusion

Therefore, this study presented the preliminary data to demonstrate the safety and effectiveness of this technique in treatment of patients with cancer spinal metastasis. These results are encouraging. Although the studied sample size was somewhat small and with the limitation mentioned above, it still was a hopeful progress in radiation therapy for patient with cancer. As a result, the application of conventionally-fractionated IG-IMRT has the potential to improve the clinical outcome of the patients with cancer spinal metastasis.

## Competing interests

The authors declare that they have no competing interests.

## Authors' contributions

YG and JW contributed equally in design of the study, collection of data and drafting the manuscript; SB and XJ worked on analysis of data; FX provided the conception of this study and the final approval of the version to be published. And all authors read and approved the final manuscript.
